# Bioaccumulation of Mercury, Cadmium, Lead, and Arsenic in Whiting and Tub Gurnard From the Sea of Marmara: Implications for Human Health

**DOI:** 10.1002/fsn3.70370

**Published:** 2025-06-01

**Authors:** Hande Dogruyol, İdil Can Tunçelli, Özkan Özden, Nuray Erkan, Firdes Saadet Karakulak

**Affiliations:** ^1^ Department of Food Safety, Faculty of Aquatic Sciences Istanbul University Istanbul Türkiye; ^2^ Department of Seafood Processing, Faculty of Aquatic Sciences Istanbul University Istanbul Türkiye; ^3^ Department of Fisheries Technology and Management, Faculty of Aquatic Sciences Istanbul University Istanbul Türkiye

**Keywords:** food safety, heavy metal, risk assessment, seafood

## Abstract

Toxic trace elements tend to accumulate in the Sea of Marmara, a semi‐enclosed body of water, exacerbating pollution and posing a threat to human health through the consumption of bottom‐dwelling fish. Mercury, cadmium, lead, and arsenic concentrations in two commercially important species, whiting (*Merlangius merlangus euxinus*) and tub gurnard (*Chelidonichthys lucerna*), were investigated from eight different locations. Considering the regulatory limits (EU Regulation 2023/915), Hg levels in whiting surpassed the safety threshold, while in tub gurnard the concentrations were higher than 0.30 g/kg at three sampling sites. None of the samples reached the limit values set for Cd and Pb. Although no official limit has been established for As in fish, its concentrations were found to be notable in both species. Overall, whiting presented a higher level of toxicological risk compared to tub gurnard. Total target hazard quotients (TTHQs) exceeded the safety threshold of 1 for individuals weighing 50 kg—typically adolescents or young adults—at all whiting sampling locations, indicating potential health risks for this sensitive population. In tub gurnard, TTHQ values surpassed 1 at only three locations. Notably, the highest TTHQ for Hg was recorded in samples from the western region of the sea, exceeding 1 for all individuals, further underscoring the potential risk associated with consumption. The carcinogenic Target Risk (TR) for inorganic As was found to be unacceptable in whiting captured from this location for all body weights and from the southern location for 50 kg people. Individuals with lower body weight are more susceptible to the risks associated with consuming demersal fish. To mitigate the risks of bioaccumulation, it is advisable to diversify frequently consumed foods.

## Graphical abstract

Mercury levels in whiting and tub gurnard captured from the Sea of Marmara exceeded EU regulatory limits. TTHQ was above 1 for individuals weighing 50, 70, and 90 kg in the western part of the sea. Consumers with lower body weights were at greater cancer risk (TR) regarding inorganic arsenic. FDA's recommendation for whiting consumption may pose a risk due to the 300 g intake.

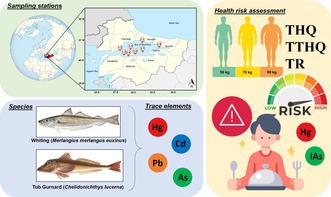

## 1 Introduction

Toxic trace elements are significant environmental and public health concerns due to their persistence and toxicity. These pollutants, whether from natural sources or anthropogenic activities, accumulate in aquatic ecosystems and subsequently in fish, posing considerable risks to human health through consumption (Ramon et al. 2021; Mehouel and Fowler 2022). They are especially challenging in regions where human activities, such as industrial waste discharge, urban runoff, and maritime transport, are intense (Liu et al. 2018; Jeong et al. 2020; Deng et al. 2021).

The limited water circulation in the Sea of Marmara exacerbates pollution and increases consumer risks. As a semi‐enclosed sea between the Mediterranean and the Black Sea, the Sea of Marmara has restricted water exchange. The low salinity of inflowing Black Sea water contrasts with the higher salinity of Mediterranean water, which primarily occupies the deeper layers (Ünlülata et al. 1990; Beşiktepe et al. 1994). Consequently, toxic trace elements accumulate in sea sediments, and hypoxic conditions promote deposition. These elements harm benthic organisms and contribute to biomagnification in fish that feed on them (Dökmeci et al. 2019a). Ecological regime shifts in the Sea of Marmara were highlighted by Demirel et al. (2023), who emphasized the impacts of human‐induced activities, overfishing, and pollution on the Sea of Marmara's marine ecosystem health. Additionally, the slow water circulation allows industrial waste and other pollutants to linger, significantly influencing metal accumulation and resulting in elevated levels of cadmium (Cd), lead (Pb), and mercury (Hg), which damage the marine ecosystem (Cucu et al. 2019).

The Sea of Marmara creates a unique habitat for various fish species. In particular, fish species such as whiting and tub gurnard are of concern due to their feeding habits and habitats, which make them prone to potentially hazardous element uptake from contaminated water and sediments. Several studies have identified fish as a major route for toxic trace elements to enter the human body through consumption, making them critical indicators of aquatic pollution (Pragnya et al. 2021). This sea is vital to Türkiye's fisheries sector, significantly contributing to the country's total fish production. It supports the livelihoods of countless fishermen and their families while sustaining related industries, including processing, distribution, and tourism. However, the sustainability of fish stocks in the Sea of Marmara faces increasing challenges from pollution, overfishing, and climate change, necessitating careful management to ensure the long‐term viability of these resources. Protecting the health of the marine ecosystem and preserving fish populations is crucial for the future of Türkiye's fisheries (Karakulak et al. 2024). Understanding the impacts of toxic trace element accumulation on commercially important species is essential for environmental conservation and public health, underscoring the need for ongoing research in this critical region.

Whiting and tub gurnard, widely consumed in the area, are key species for assessing toxic trace element bioaccumulation. Research indicates that marine organisms, especially bottom‐dwelling species like gurnard, are highly susceptible to potentially hazardous element accumulation due to their feeding behaviors and habitats. As bioindicators, their size, lifespan, trophic level, and role in human consumption make them effective for monitoring aquatic pollution, with trace elements from benthic organisms transferring through the food chain and impacting human health (Zhao et al. 2012; Cabrini et al. 2018). Toxic trace elements can present health risks to humans, such as the ingestion of Pb, which can lead to developmental and neurological issues, especially in children, and Cd, primarily from industrial pollution, which can cause kidney damage and weaken bones over time. Similarly, Hg, particularly in its organic form methylmercury, poses neurotoxic risks when bioaccumulated in fish (Ahmed et al. 2019). Thus, accurately evaluating the health risks associated with consuming seafood contaminated with toxic elements is crucial.

Based on previous research (Capodiferro et al. 2022; Ulusoy et al. 2025), we hypothesize that whiting may exhibit higher Hg bioaccumulation than tub gurnard, leading to elevated health risks. This study aimed to quantify the concentrations of toxic trace elements such as Hg, Pb, Cd, and As in whiting and tub gurnard collected from different locations in the Sea of Marmara. Potential health risks to human health were also investigated within a comprehensive assessment. THQ, TTHQ, and TR were calculated for female and male populations weighing 50, 70, or 90 kg that include adolescent, adult, and elder age groups.

## 2 Materials and Methods

### 2.1 Study Area and Sampling

**FIGURE 1:**
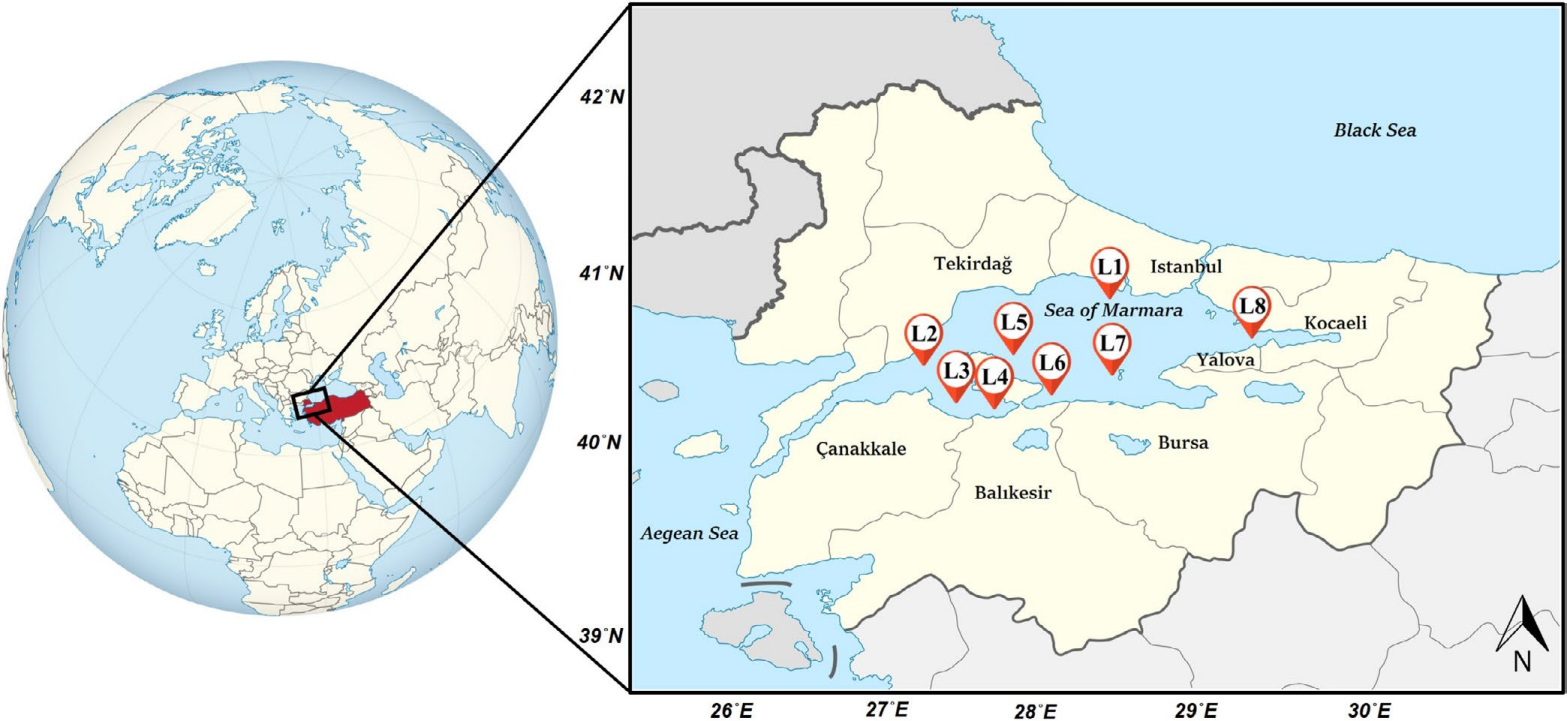
Map of the study area where whiting and tub gurnard were collected in the Sea of Marmara.

Whiting (*Merlangius merlangus euxinus* Nordmann, 1840) and tub gurnard (*Chelidonichthys lucerna* Linnaeus, 1758) were captured from various regions of the Sea of Marmara during the autumn of 2019 by Istanbul University's research ship “Yunus‐S.” The sampling was conducted at multiple locations, labeled L1 through L8 (Figure 1), representing different regions across the Sea of Marmara. These locations covered diverse marine environments, including coastal and deeper offshore zones. Specific details about the geographic coordinates of the sampling sites, trawling depths, and the number of samples collected from each species are presented in Table 1.

**TABLE 1:** Coordinates, trawling depths, and collection overview of sampling sites L1–L8 in the Sea of Marmara.

Species	Coordinates	Trawling depths (m)	Station	Length (cm)	Weight (g)	*n*
Whiting	40°57′29.46″N 28°23′49.43″ E	75	L1	25.78 ± 2.61	138.88 ± 47.06	6
	40°37′05.00″N 27°24′19.00″ E	108	L2	26.50 ± 4.22	139.81 ± 79.19	7
	40°25′25.00″N 27°31′03.00″ E	45	L3	14.95 ± 1.12	26.20 ± 6.28	42
	40°38′31.00″N 27°50′38.00″ E	62	L5	34.20 ± 2.49	310.98 ± 85.88	5
	40°24′12.44″N 28°05′17.55″ E	46	L6	13.66 ± 0.87	18.71 ± 3.95	69
	40°32′15.00″N 28°25′50.00″ E	46	L7	14.97 ± 1.96	29.70 ± 14.19	65
Tub gurnard	40°57′29.46″N 28°23′49.43″ E	75	L1	26.58 ± 3.28	189.80 ± 80.39	5
	40°25′25.00″N 27°31′03.00″ E	45	L3	19.30 ± 2.99	69.42 ± 34.67	5
	40°21′20.00″N 27°47′13.00″ E	37	L4	23.26 ± 5.22	141.74 ± 113.15	5
	40°38′31.00″N 27°50′38.00″ E	62	L5	30.00 ± 4.59	266.20 ± 135.49	5
	40°24′12.44″N 28°05′17.55″ E	46	L6	24.83 ± 4.45	170.67 ± 99.27	9
	40°44′45.00″N 29°19′58.00″ E	65	L8	33.42 ± 9.61	338.40 ± 291.90	5

*Note: n*: number of the individuals captured from each location. Weight and length of the samples were given as Mean ± Standard Deviation.

The trawling process lasted for 30 min, at a speed of 3 miles/h, covering an area of approximately 0.03 km^2^. Immediately after collection, the fish were frozen at −20°C to maintain the integrity of the samples. The frozen samples were transported to the laboratory in polystyrene containers, ensuring the cold chain remained intact throughout the journey. After being brought to the laboratory, the fish were processed by removing their heads and internal organs. Whiting and tub gurnard fillets were then homogenized (Retsch, GM200, Germany) to prepare them for further analysis for toxic trace element concentration assessment. For each sampling station (L1–L8), fillets from multiple individuals of the same species were pooled to create a composite sample representative of that location. All fish length measurements refer to total length (TL). The total of all captured individuals of a species from each specific location were given in Table 1.

### 2.2 Toxic Trace Element Analysis

The homogenized samples, weighing between 0.1 and 0.2 g, were first rinsed with distilled water. They were processed (for digestion) using a microwave digestion system (MWS‐4, Berghof, Germany) after adding a mixture of hydrogen peroxide and nitric acid in a 1:2 ratio. The digestion method followed the procedure outlined by Guhathakurta and Kaviraj (2000) and was performed in duplicate. After digestion, the extracts were transferred to 50 mL sample tubes to prepare them for analysis using inductively coupled plasma mass spectrometry (ICP‐MS; ELAN‐DRC‐e, Perkin Elmer, USA). Each set of samples included a blank sample and certified reference material (ERM‐BB422 Fish muscle). The recovery rates for arsenic (As), cadmium (Cd), and mercury (Hg) from the reference material were 104.9%, 96.1%, and 98.7%, respectively. Calibration standards were prepared from a 10,000 μg/kg stock solution of the analyzed elements, with concentrations set at 0.2, 0.5, 1, 5, 10, 20, 50, 100, and 200 μg/kg for Hg, Cd, Pb, and As. The limits of detection (LODs) for these elements were 0.004 μg/kg for As, 0.002 μg/kg for Cd, 0.001 μg/kg for Pb, and 0.005 μg/kg for Hg. Trace element analyses were conducted on a wet weight (ww) basis, and the results were presented as mg/kg.

### 2.3 Assessment of Potential Health Risks

This study conducted a comprehensive health risk assessment concerning toxic element exposure from fish consumption, focusing on whiting and tub gurnard. The key indicators utilized in the analyses included the Target Hazard Quotient (THQ) and Target Carcinogenic Risk (TR). Health risks exhibit significant variation across gender groups, as demonstrated by the projected life expectancy at birth in 2024, which is 71 years for males and 76 years for females. According to the United Nations Population Fund (2024), average body weights of 50, 70, and 90 kg have been categorized by gender groups. In order to lower the risk of developing acute coronary syndromes, the recommended weekly fish consumption rate (weekly ingestion rate) is 150 g (Panagiotakos et al. 2005). The Food and Drug Administration (FDA 2024) advises consuming seafood at least 8 oz (~227 g)/week based on a 2000 cal diet, and 8–12 oz (~340 g) per week for pregnant or breastfeeding females. Therefore, the average fish consumption rate was regarded as 300 g per week‐capita in this study.

### 2.4 Risk Evaluation Using THQ and TR

The health risks associated with fish consumption were evaluated using THQ and TR metrics. THQ measures non‐carcinogenic risk due to toxic trace element exposure, while TR estimates the lifetime risk of developing cancer from contaminant exposure. The assessment assumed that inorganic arsenic (iAs) accounted for 10% of the total As content, representing the more toxic fraction. For mercury, it was assumed that total Hg was primarily in the form of methylmercury (MeHg), which is known to be highly toxic (Qin et al. 2015; Özden et al. 2020).

The equations for calculating THQ and TR were adapted from the US EPA (2023a):

- Target Hazard Quotient (THQ):

(1) $$\mathrm{THQ}=0.001*\frac{\mathrm{EF}*\mathrm{ED}*\mathrm{IFR}*\mathrm{Cm}}{\mathrm{BW}*\mathrm{AT}*\mathrm{RfD}}$$

- Target Risk (TR):

(2) $$\mathrm{TR}=0.001*\frac{\mathrm{EF}*\mathrm{ED}*\mathrm{IFR}*\mathrm{Cm}*\mathrm{CSF}}{\mathrm{BW}*\mathrm{AT}}$$

### 2.5 Parameters for Health Risk Assessment

This comprehensive health risk assessment utilized several critical parameters:

- **EF**: Exposure frequency, set at 350 days per year,
- **ED**: Exposure duration (years),
- **IFR**: Food ingestion rate (g/day),
- **Cm**: Trace element concentration (mg/kg) in fish,
- **CSF**: Oral carcinogenic slope factor for assessing cancer risk,
- **BW**: Body weight (kg),
- **AT**: Average exposure time for non‐carcinogenic effects, calculated as 365 days/year over 70 years,
- **RfD**: Oral reference dose (mg/kg/day) representing the safe exposure level for each element.

The reference values for RfD were:

- **Mercury** (**Hg**): 0.0001 mg/kg/day,
- **Cadmium** (**Cd**): 0.0001 mg/kg/day,
- **Inorganic Arsenic** (**iAs**): 0.0003 mg/kg/day (US EPA 2023b).

The CSF values for evaluating carcinogenic risk were:

- **Lead** (**Pb**): 8.5 × 10^−3^ (mg/kg‐day)^−1^,
- **Inorganic Arsenic** (**iAs**): 1.5 (mg/kg‐day)^−1^ (US EPA 2023b).

Based on data from the United States Environmental Protection Agency (US EPA 2023b) for life expectancy, the exposure duration (ED) was 26 years.

To ensure consumer safety, THQ values were calculated for Hg, Cd, and iAs, and TTHQ was determined. For Pb, US EPA (2004) concluded that it is inappropriate to determine an RfD due to its adverse health effects. Hence, THQ for Pb was not evaluated. Additionally, TR was calculated for Pb and iAs.

### 2.6 Statistical Analysis

All statistical analyses and data visualizations were conducted using *R* software (version 4.4.2) and IBM SPSS Statistics (version 29). Prior to hypothesis testing, the normality of each element concentration (As, Cd, Pb, Hg) was assessed through both visual methods (histograms and Q‐Q plots) and the Shapiro–Wilk test. Results indicated non‐normal distributions for As, Cd, and Hg (*p* < 0.05), while Pb showed a normal distribution (*p* = 0.32). Accordingly, non‐parametric Mann–Whitney *U* tests were applied to compare As, Cd, and Hg concentrations between fish species (whiting and tub gurnard), whereas Pb was analyzed using an independent samples *t*‐test.

To evaluate spatial differences across eight sampling locations (L1–L8), Kruskal–Wallis tests were used for As, Cd, and Pb, and Welch's ANOVA was applied for Hg due to unequal variances. When significant differences were found, post hoc tests were conducted (Dunn's test with Bonferroni correction for Kruskal–Wallis; Games–Howell test for Welch's ANOVA). Furthermore, a two‐way ANOVA was employed to assess the interaction effects of species and location for each element, with significance thresholds set at *p* < 0.05. Given the presence of substantial missing data (particularly for Pb, with 68.1% missing), no imputation was performed. Instead, each element was analyzed independently based on available data. Only 19.1% of the samples had complete measurements for all four elements. Triplicate measurements were summarized as mean ± standard deviation.

Additionally, a normalized composite contamination index was constructed to integrate all four elements and assess the overall contamination burden by species and location. Finally, Principal Component Analysis (PCA) was performed in *R* to visualize multivariate patterns in toxic trace element profiles and to explore clustering trends by species and sampling site. The first two principal components explained over 80% of the total variance. Biplots were generated with 95% confidence ellipses to illustrate species‐related grouping tendencies.

## 3 Results and Discussion

### 3.1 Toxic Trace Element Concentrations

**FIGURE 2:**
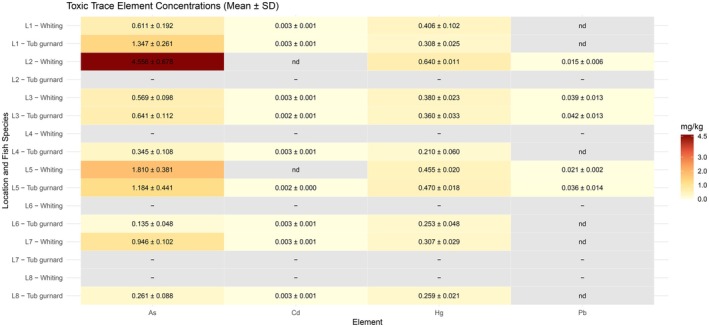
Toxic trace element concentrations in whiting and tub gurnard species captured from different locations (L1–L8). Nd, not in detectable limits (limits of detection Cd: 0.002; Pb: 0.001 μg/kg); ±, standard deviation; −, the species was not captured at the location.

Toxic trace elements such as mercury, cadmium, lead, and arsenic are a cause for concern in aquatic habitats. They are toxic to all organisms and have no biological tasks. Besides, they have the potential for serious adverse health effects on humans when ingested (WHO 1980; Chouvelon et al. 2012). The concentrations of toxic trace elements in whiting and tub gurnard, captured from different locations, are presented in Figure 2.

Prior to interspecies comparisons, the normality of trace element data (As, Cd, Pb, Hg) was evaluated using the Shapiro–Wilk test and visual inspection (histograms and Q–Q plots). Arsenic (As), cadmium (Cd), and mercury (Hg) significantly deviated from normal distribution (*p* < 0.05), whereas lead (Pb) was normally distributed (*p* = 0.32). Accordingly, Mann–Whitney *U* tests were used for As, Cd, and Hg, while Pb was analyzed using an independent samples *t*‐test.

The results revealed significant interspecies differences for As and Hg, with whiting exhibiting higher mean concentrations of both elements compared to tub gurnard. Arsenic levels were significantly higher in whiting (mean: 1.69 μg/g) than in tub gurnard (mean: 0.60 μg/g; *p* = 0.0009). Similarly, Hg concentrations were higher in whiting (mean: 0.43 μg/g) compared to tub gurnard (mean: 0.29 μg/g; *p* = 0.0007). In contrast, no significant difference was observed in Cd levels (*p* = 0.355), with both species exhibiting consistently low values (< 0.003 μg/g). For Pb, a borderline significant difference was detected (*p* = 0.050), with tub gurnard showing slightly higher mean values; however, this finding is limited by the high proportion of missing Pb data (68.1%).

To evaluate spatial differences across the eight sampling locations (L1–L8), non‐parametric Kruskal–Wallis tests were applied for As, Cd, and Pb, and Welch's ANOVA was used for Hg. Arsenic and Hg exhibited highly significant spatial variation (*p* < 0.0001 for both), with the highest concentrations observed at location L2 (As: 4.56 μg/g; Hg: 0.64 μg/g) and the lowest at L6 and L4, respectively. Post hoc analyses (Dunn's and Games–Howell tests) identified multiple significant pairwise differences among locations. Pb also showed a significant location effect (*p* = 0.028), though limited by data availability. Cd did not vary significantly across locations (*p* = 0.703), remaining consistently low.

Two‐way ANOVA revealed a significant interaction between species and location for As (*p* = 0.015), suggesting that the magnitude of species differences varied across sites. For instance, while whiting had markedly higher As at L2, tub gurnard had relatively elevated As at L1. A marginal interaction effect was also noted for Hg (*p* = 0.061), again indicating location‐dependent interspecies variation. These findings highlight the complex interplay between ecological context and bioaccumulation patterns.

**FIGURE 3:**
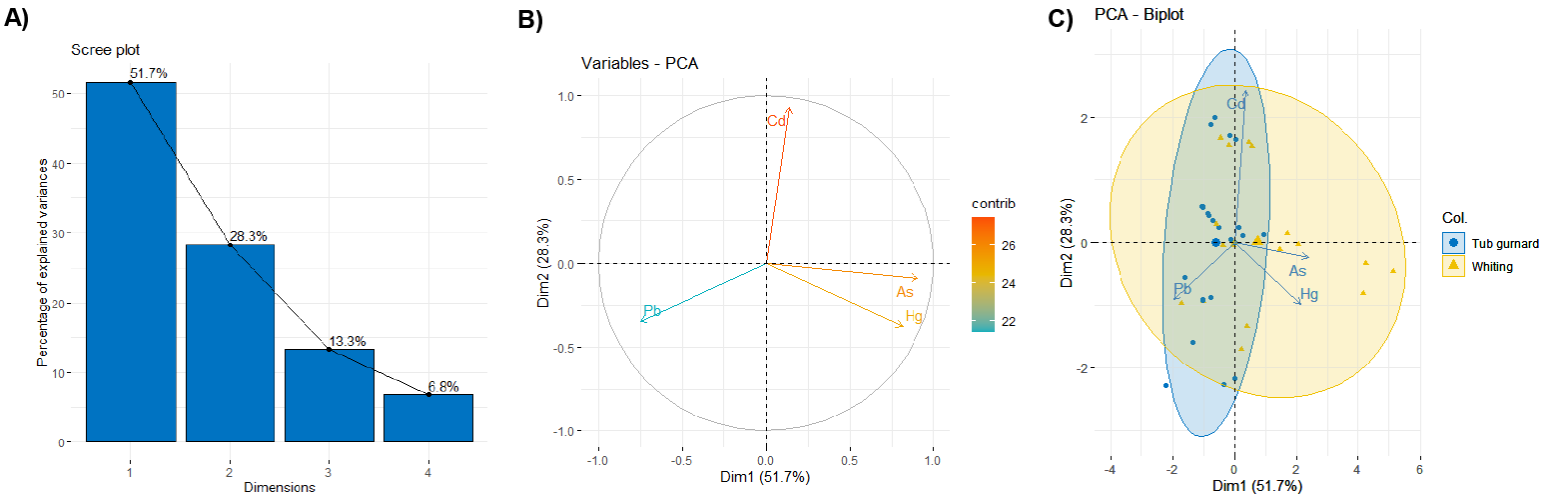
Principal component analysis (PCA) of trace elements in fish species: (A) Scree plot. (B) Loading plot. (C) Biplot with species clustering.

Principal Component Analysis (PCA) was conducted to explore overall patterns in toxic trace element profiles across species and locations (Figure 3). The first two principal components (PC1 and PC2) explained a cumulative 80.0% of total variance (51.7% and 28.3%, respectively) (Figure 3A,B). As shown in the loading plot, As and Hg strongly aligned with PC1, while Cd dominated PC2. Pb contributed minimally to the total variance due to its limited presence.

In the PCA biplot (Figure 3C), whiting samples clustered distinctly in the direction of As and Hg, reflecting their higher contamination levels, whereas tub gurnard samples showed partial association with Cd. These multivariate trends are consistent with the univariate findings and reinforce the notion of species‐specific and location‐dependent accumulation profiles.

Tub gurnard samples clustered more closely with Cd, possibly due to their benthic lifestyle and greater contact with sediment‐bound contaminants. In contrast, whiting samples aligned more with Hg and As, likely reflecting trophic level differences or dietary intake. These patterns suggest underlying ecological and physiological factors influencing species‐specific accumulation.

For whiting, the maximum level for Hg is set at 0.30 mg/kg as stated in EU Commission Regulation 2023/915 (European Commission 2023). The Hg concentrations were between 0.307 and 0.640 mg/kg in whiting, which were higher than the threshold level. The mean concentrations of Hg measured in our whiting samples obtained from locations L1, L2, and L3 were found to be higher than the samples captured from Saros and Edremit Bays, Aegean Sea (Dogruyol et al. 2023). After the flood incident on the coasts of southern Black Sea, Duyar et al. (2023) determined that the Hg levels in whiting significantly raised from 0.05 to 0.15 mg/kg and fortunately remained under the limit. These Hg concentrations were much lower than those in the samples from the Sea of Marmara. On the other hand, the allowed concentration for fish species that are not directly addressed is 0.50 mg/kg. Considering the same sampling locations as whiting, the Hg levels in tub gurnard exceeded 0.30 mg/kg at locations L1, L3, and L5. Ulusoy et al. (2025) reported that the mean Hg concentration was 0.299 mg/kg in tub gurnard from the Sea of Marmara, which was below the limit. Similar Hg levels (0.328 mg/kg) in tub gurnard captured from Saros Bay of Aegean Sea were found when compared to locations L1 and L3, while lower values were observed in regions L4 and L8 (Dogruyol et al. 2023). Elevated concentrations at particular regions demonstrate that there might be a high input of anthropogenic influences. Consistent with the concentrations observed in species captured from L1 to L5, Chouvelon et al. (2012) also reported that the tub gurnard captured from Biscay Bay, NE Atlantic, had higher levels of Hg (0.964–1.411 mg/kg). On the other hand, Hg levels of tub gurnard caught from Portuguese coastal seawaters were below the permissible limit (0.020–0.104 mg/kg) (Reis et al. 2020).

The cadmium concentrations in both whiting and tub gurnard were found between 0.002 and 0.003 mg/kg. At L2 and L5 locations, the Cd level was below the detection limit in whiting. Since the permissible limit for both species is 0.050 mg/kg (European Commission 2023), Cd levels were well below this value. In line with our study, similar Cd levels were reported in tub gurnard, and non‐detectable levels of Cd were observed in whiting from Saros Bay (Dogruyol et al. 2023). Regarding Pb, the permissible limit is 0.30 mg/kg in muscle meat of fish (European Commission 2023). In this study, none of the samples at any location reached that high value, and they remained under the detection limit at L1, L4, L6, L7, and L8 where the fish were captured. There is no maximum limit for arsenic, except for various rice foods and baby foods (European Commission 2023). In this research, the As concentrations were between 0.569 and 4.556 mg/kg in whiting and 0.135–1.347 mg/kg in tub gurnard.

In the literature, various studies on toxic elements in whiting and tub gurnard have yielded different results based on the region. Cucu et al. (2019) detected lower concentrations of Cd and Pb in whiting from the Gebze region of the Sea of Marmara with the maximum amounts of 0.000263 and 0.00225 mg/kg, respectively, in comparison to our study. From the central Black Sea (Rize) mean Pb was found to be 0.015 mg/kg, equal to our L2 sampling area (Şirin et al. 2024). In another investigation from the Black and Aegean Seas, the levels in the same species were higher than those in our study, which were 0.55 mg/kg for Cd and 0.93 mg/kg for Pb (Uluozlu et al. 2007). Differing from our study, Türkmen et al. (2008) observed higher Cd (0.02 mg/kg) and Pb (0.33 mg/kg) levels in gurnard samples (*Trigla gurnardus*) from the Yalova region of the Sea of Marmara. Also, higher mean Cd and Pb concentrations (0.40 and 6.80 mg/kg) were detected in whiting from the Bartin region of the western Black Sea (Fındık and Çiçek 2011). Unlikely, Mol et al. (2017) detected higher mean concentrations of Cd (0.02 mg/kg) and Pb (0.360 mg/kg), but lower As value (4.09 mg/kg) in whiting captured from the SW Black Sea, in comparison to our study. Another elevated value of Cd (0.031 mg/kg) and As (6.34 mg/kg) were observed from the Ordu and Samsun regions of the Black Sea (Alkan et al. 2016).

The concentrations of Pb and As in the tub gurnard from Saros Bay, Aegean Sea, were 0.043 and 0.574 mg/kg, respectively (Dogruyol et al. 2023). The Cd levels of 0.04–0.07 mg/kg and Pb levels of 0.12–0.42 mg/kg were detected from Iskenderun Bay, located in the eastern Mediterranean (Ersoy and Çelik 2010). In another study in the same bay, Cd, Pb, and As levels in tub gurnard were 0.01, 0.14, and 1.38 mg/kg, respectively (Yılmaz et al. 2010). Increased Cd and Pb concentrations were determined, namely 0.46 and 1.26 mg/kg, in the samples captured from Iskenderun Bay. However, lower values were obtained from the Yalova region of the Sea of Marmara (0.01 mg/kg for Cd; 0.27 mg/kg for Pb) (Ateş et al. 2015). Bat et al. (2017) stated that the Cd, Hg, and Pb in tub gurnard caught from Sinop coasts, central Black Sea, were below the limit of detection. Tub gurnard from Portuguese NW coastal seawaters had Cd levels of 0.002–0.089 mg/kg and Pb levels of 0.0048–0.403 mg/kg dry weight (Reis et al. 2020). Depending on the region and anthropogenic activities, various results have been observed in numerous studies regarding tub gurnard. Furthermore, it is important to acknowledge that aquatic organisms are often exposed to multiple toxic elements simultaneously. The interactions between these elements can result in additive, antagonistic, or even synergistic effects, which may enhance toxicity beyond the individual impact of each element (Adeleye et al. 2024). Thus, evaluating only single‐element contamination may underestimate the potential environmental risk posed by the combined presence of trace elements in marine ecosystems.

### 3.2 Potential Health Risk Assessment

Toxic trace elements' levels in sediment may be relatively low; however, even small amounts can directly affect the benthic organisms. Toxic trace elements like Hg are known for biomagnifying through food chains, and others like Cd, Pb, and As in the habitat contribute to bioaccumulation. This route starts from aquatic microorganisms, macro algae (Önel et al. 2025), and shellfish (Önel et al. 2024) to fish (Kalipci et al. 2023) and eventually ends with humans. Therefore, evaluating potential health risks across different consumer groups is substantial in research on toxic element quantification and monitoring.

Consumers who are particularly uncomfortable with strong fishy odors may find a lean, white‐fleshed, mild‐flavored, freshly filleted product more appealing (Murray et al. 2017). Moreover, children particularly prefer boneless white flesh fillets (McManus et al. 2007). According to the FDA (2024), whiting is considered a healthy choice because of its low mercury content. It is recommended to eat 2–3 servings per week of whiting and certain fish species (from the Best Choices list) during pregnancy and breastfeeding. Since one serving is determined as 113.4 g (4 oz), the weekly recommended portion is between ~227 and 340 g (8–12 oz) (FDA 2024). Therefore, the average weekly consumption amount was considered to be 300 g in this study.

Health risk calculations are crucial to identify potential hazards, take preventive measures, and promote healthy individuals and society. They play a critical role in managing risks and maintaining food security (Erkan 2023). Target Hazard Quotient (THQ) is the non‐carcinogenic health risk index related to exposure to a toxic substance during a lifetime. The THQ values above 1 imply a potential adverse effect on health (Ulusoy et al. 2025). On the other hand, TR is the estimated cancer risk index resulting from lifelong exposure to a contaminant. The TR index can be calculated for pollutants with a known cancer slope factor. This approach to risk management acknowledges that a range of one‐in‐a‐million (10^−6^) to one‐in‐ten‐thousand (10^−4^) may be “acceptable” for carcinogenic risk. Values greater than 10^−4^ indicate that the risk arising from a certain substance is unacceptable (US EPA 2024).

Higher THQ values for all toxic elements in both whiting and tub gurnard indicated higher potential non‐carcinogenic health risks. In this study, no risks were noted in the samples according to the THQ calculations of Cd, Pb, and iAs (Tables 2 and 3). On the other hand, THQ‐Hg was higher than 1 at every sampling location in whiting, and at L1, L3, and L5 in tub gurnard samples for 50 kg individuals who are more likely to be adolescents or underweight adults. Highest risks were observed for all body weights and genders in whiting from location L2 where the Hg concentration was the highest. Similarly, the THQ‐Hg index of whiting obtained from Edremit Bay, northern Aegean Sea, was above the threshold for the seven different age categories calculated for the weights ranging between 14 and 77 kg. Also, the THQ‐As exceeded 1 for the individuals weighing between 14 and 51 kg. TTHQ values also surpassed the limit for all age groups as well, and the TR index for iAs indicated unacceptable risk for 72–77 kg consumers (Dogruyol et al. 2023). In contrast to our study, Mol et al. (2017) reported that the THQ values of Cd, Hg, and Pb and their TTHQ of whiting caught from the SW Black Sea were below 1 and did not pose any health risks. Şirin et al. (2024) pointed to an absence of risk according to THQ, TTHQ, and TR calculations for whiting from the eastern Black Sea.

**TABLE 2:** Target Hazard Quotient (THQ), Total THQ (TTHQ), and Target Carcinogenic Risk (TR) calculations of related elements in whiting captured from seven different locations according to 50, 70, and 90 kg male and female individuals.

Location	Human		Whiting					
			THQ				TR	
	Gender	Weight (kg)	Hg	Cd	iAs	TTHQ	Pb	As
L1	Male	50	**1.222**	0.009	0.061	**1.292**	0.00E+00	2.76E‐05
		70	0.873	0.006	0.044	0.923	0.00E+00	1.97E‐05
		90	0.679	0.005	0.034	0.718	0.00E+00	1.53E‐05
	Female	50	**1.142**	0.008	0.057	**1.207**	0.00E+00	2.58E‐05
		70	0.815	0.006	0.041	0.862	0.00E+00	1.84E‐05
		90	0.634	0.005	0.032	0.671	0.00E+00	1.43E‐05
L2	Male	50	**1.926**	0.000	0.457	**2.383**	3.84E‐08	**2.06E‐04**
		70	**1.376**	0.000	0.326	**1.702**	2.74E‐08	**1.47E‐04**
		90	**1.070**	0.000	0.254	**1.324**	2.13E‐08	**1.14E‐04**
	Female	50	**1.800**	0.000	0.427	**2.227**	3.59E‐08	**1.92E‐04**
		70	**1.285**	0.000	0.305	**1.590**	2.56E‐08	**1.37E‐04**
		90	**1.000**	0.000	0.237	**1.237**	1.99E‐08	**1.07E‐04**
L3	Male	50	**1.144**	0.009	0.057	**1.210**	9.98E‐08	2.57E‐05
		70	0.817	0.006	0.041	0.864	7.13E‐08	1.83E‐05
		90	0.635	0.005	0.032	0.672	5.54E‐08	1.43E‐05
	Female	50	**1.068**	0.008	0.053	**1.130**	9.32E‐08	2.40E‐05
		70	0.763	0.006	0.038	0.807	6.66E‐08	1.71E‐05
		90	0.594	0.005	0.030	0.628	5.18E‐08	1.33E‐05
L5	Male	50	**1.369**	0.000	0.182	**1.551**	5.37E‐08	8.17E‐05
		70	0.978	0.000	0.130	**1.108**	3.84E‐08	5.84E‐05
		90	0.761	0.000	0.101	0.862	2.98E‐08	4.54E‐05
	Female	50	**1.279**	0.000	0.170	**1.449**	5.02E‐08	7.63E‐05
		70	0.914	0.000	0.121	**1.035**	3.59E‐08	5.45E‐05
		90	0.711	0.000	0.094	0.805	2.79E‐08	4.24E‐05
L6	Male	50	**1.345**	0.003	0.282	**1.630**	5.37E‐08	**1.27E‐04**
		70	0.961	0.002	0.201	**1.165**	3.84E‐08	9.06E‐05
		90	0.747	0.002	0.157	0.906	2.98E‐08	7.05E‐05
	Female	50	**1.257**	0.003	0.263	**1.523**	5.02E‐08	**1.19E‐04**
		70	0.898	0.002	0.188	**1.088**	3.59E‐08	8.47E‐05
		90	0.698	0.002	0.146	0.846	2.79E‐08	6.58E‐05
L7	Male	50	0.924	0.009	0.095	**1.028**	0.00E+00	4.27E‐05
		70	0.660	0.006	0.068	0.734	0.00E+00	3.05E‐05
		90	0.513	0.005	0.053	0.571	0.00E+00	2.37E‐05
	Female	50	0.863	0.008	0.089	0.960	0.00E+00	3.99E‐05
		70	0.617	0.006	0.063	0.686	0.00E+00	2.85E‐05
		90	0.480	0.005	0.049	0.534	0.00E+00	2.22E‐05

*Note:* Bold values indicate exceedance of the specified limit.

**TABLE 3:** Target Hazard Quotient (THQ), Total THQ (TTHQ), and Target Carcinogenic Risk (TR) calculations of related elements in tub gurnard captured from eight different locations according to 50, 70, and 90 kg male and female individuals.

Location	Human		Tub gurnard					
			THQ				TR	
	Gender	Weight (kg)	Hg	Cd	iAs	TTHQ	Pb	As
L1	Male	50	0.927	0.009	0.135	**1.071**	0.00E+00	6.08E‐05
		70	0.662	0.006	0.097	0.765	0.00E+00	4.34E‐05
		90	0.515	0.005	0.075	0.595	0.00E+00	3.38E‐05
	Female	50	0.866	0.008	0.126	**1.001**	0.00E+00	5.68E‐05
		70	0.619	0.006	0.090	0.715	0.00E+00	4.06E‐05
		90	0.481	0.005	0.070	0.556	0.00E+00	3.16E‐05
L3	Male	50	**1.144**	0.006	0.036	**1.186**	1.07E‐07	1.63E‐05
		70	0.817	0.004	0.026	0.847	7.68E‐08	1.16E‐05
		90	0.635	0.003	0.020	0.659	5.97E‐08	9.03E‐06
	Female	50	**1.068**	0.006	0.034	**1.108**	1.00E‐07	1.52E‐05
		70	0.763	0.004	0.024	0.791	7.17E‐08	1.08E‐05
		90	0.594	0.003	0.019	0.615	5.58E‐08	8.44E‐06
L4	Male	50	0.632	0.009	0.035	0.676	0.00E+00	1.56E‐05
		70	0.451	0.006	0.025	0.483	0.00E+00	1.11E‐05
		90	0.351	0.005	0.019	0.375	0.00E+00	8.65E‐06
	Female	50	0.590	0.008	0.032	0.631	0.00E+00	1.46E‐05
		70	0.422	0.006	0.023	0.451	0.00E+00	1.04E‐05
		90	0.328	0.005	0.018	0.351	0.00E+00	8.08E‐06
L5	Male	50	**1.369**	0.006	0.047	**1.423**	9.21E‐08	2.12E‐05
		70	0.978	0.004	0.034	**1.016**	6.58E‐08	1.52E‐05
		90	0.761	0.003	0.026	0.790	5.12E‐08	1.18E‐05
	Female	50	**1.279**	0.006	0.044	**1.329**	8.60E‐08	1.98E‐05
		70	0.914	0.004	0.031	0.949	6.15E‐08	1.42E‐05
		90	0.711	0.003	0.024	0.738	4.78E‐08	1.10E‐05
L6	Male	50	0.761	0.009	0.014	0.784	0.00E+00	6.09E‐06
		70	0.544	0.006	0.010	0.560	0.00E+00	4.35E‐06
		90	0.423	0.005	0.008	0.436	0.00E+00	3.39E‐06
	Female	50	0.711	0.008	0.013	0.732	0.00E+00	5.69E‐06
		70	0.508	0.006	0.009	0.523	0.00E+00	4.07E‐06
		90	0.395	0.005	0.007	0.407	0.00E+00	3.16E‐06
L8	Male	50	0.780	0.009	0.026	0.815	0.00E+00	1.18E‐05
		70	0.557	0.006	0.019	0.582	0.00E+00	8.42E‐06
		90	0.433	0.005	0.015	0.453	0.00E+00	6.55E‐06
	Female	50	0.728	0.008	0.024	0.761	0.00E+00	1.10E‐05
		70	0.520	0.006	0.017	0.544	0.00E+00	7.86E‐06
		90	0.405	0.005	0.014	0.423	0.00E+00	6.12E‐06

*Note:* Bold values indicate exceedance of the specified limit.

According to the health risk assessment for a 70 kg individual, Dökmeci et al. (2019b) reported that the THQ index calculated for iAs in whiting from the Tekirdağ region of the Sea of Marmara was 4.7. On the coasts of the southern Black Sea, the Hg contamination in whiting significantly increased after a flood incident, posing a health threat for the 70 kg individuals with a THQ‐Hg level higher than 1. At the same samples, the TR index calculated for As indicated unacceptable risk with higher values than 10^−4^ both before and after the incident (Duyar et al. 2023). It is important to note that both studies calculated THQ and TR indices according to earlier US EPA (1989) formulae. In this study, the TR of iAs was found to be unacceptable for male and female individuals weighing 50, 70, and 90 kg for whiting samples collected from location L2. Moreover, location L6 also posed a risk for 50 kg individuals (Table 2).

Similar to our study, the THQ index of Hg calculated for tub gurnard captured from Saros Bay, northern Aegean Sea, was above 1 for the consumers weighing between 14 and 67 kg. Also, the TTHQ index was determined to be higher than 1 for up to 72 kg individuals. On the other hand, the TR index showed no cancer risk for either Pb or iAs in this study (Table 3). Similarly, Dogruyol et al. (2023) stated that tub gurnard did not pose any risks in terms of TR indices of Pb and iAs. In contrast, Ulusoy et al. (2025) reported no risks in terms of THQ‐Hg and hazard index for the consumers weighing 50, 60, and 70 kg for tub gurnard from the Sea of Marmara. Yet it is important to emphasize that the youngest (with lower body weight) and the oldest (with longer lifetime exposure) groups in the population are affected more than others (Vieira et al. 2011). Bat et al. (2017) also revealed that consuming tub gurnard from Sinop coasts, central Black Sea did not pose any risks because of the relatively unpolluted coastline. In another study, the THQ of Hg, Cd, and Pb calculated for tub gurnard from the Adriatic Sea were 0.26, 0.01, and 0.003, respectively. In addition, the TTHQ index was determined as 0.27, indicating no health risk (Storelli 2008). Similar to the risks posed by individual elements, the potential health impacts of simultaneous exposure to multiple contaminants must also be evaluated. Even when the concentration of each trace element in fish remains within acceptable limits, their combined presence may lead to chronic health effects over time due to additive or synergistic interactions (Jaishankar et al. 2014). This concern is especially relevant for vulnerable groups, including children, pregnant individuals, and those who consume seafood frequently. Incorporating such temporal data into future risk assessments would provide a more comprehensive understanding of the persistence and progression of environmental pollution.

## 4 Conclusion

The significant variations in toxic trace element concentrations among different locations in the Sea of Marmara provide insight regarding the environmental pollution and seafood safety. The mercury (Hg) levels in whiting were detected to be above the limits set by EU Commission regulations. Tub gurnard also exceeded the safety threshold for Hg contamination at some locations. Predominantly, consumers are exposed to more than one contaminant when ingesting bottom‐dwelling fish. Therefore, it is important to sum all the target hazard quotients (THQs) associated with each element. THQs and TTHQs of Hg calculated for whiting exceeded the safety threshold for 50 kg individuals at every sampling location, and for all body weights at the western location of the sea. Based on 300 g/week whiting consumption, the FDA (2024)'s recommended fish intake elevates health risks in terms of Hg, particularly in some regions of the Sea of Marmara. It is also essential to calculate health risks for pollutants that have adverse effects on humans, such as inorganic arsenic, which has no limit value for seafood, but has a cancer slope factor. It was determined that iAs may pose a carcinogenic risk in underweight individuals at L6 and all body weights at L2. Seasonal variations in contamination levels and the cumulative impact of different elements over time underscore the complexity of assessing contamination risks. The need for further studies on other commercially relevant species has been emphasized in this area. For sustainable sources and healthier seafood for well‐being of public, effective pollution control measures should be prioritized by the authorities and lawmakers.

## Author Contributions

**Hande Dogruyol:** conceptualization (equal), investigation (equal), writing – review and editing (equal). **İdil Can Tunçelli:** formal analysis (equal), methodology (equal), visualization (equal), writing – original draft (equal). **Nuray Erkan:** conceptualization (equal), supervision (equal). **Firdes Saadet Karakulak:** resources (equal). **Özkan Özden:** formal analysis (equal), methodology (equal), writing – original draft (equal).

## Conflicts of Interest

The authors declare no conflicts of interest.

## Data Availability

The data presented in the manuscript are available on request from the corresponding author.
